# Kinetic Controlled Tag-Catcher Interactions for Directed Covalent Protein Assembly

**DOI:** 10.1371/journal.pone.0165074

**Published:** 2016-10-26

**Authors:** Lee Ling Tan, Shawn S. Hoon, Fong T. Wong

**Affiliations:** Molecular Engineering Lab, Biomedical Sciences Institutes, Biopolis Drive, Singapore, Singapore; United States Army Medical Research Institute of Infectious Diseases, UNITED STATES

## Abstract

Over the last few years, a number of different protein assembly strategies have been developed, greatly expanding the toolbox for controlling macromolecular assembly. One of the most promising developments is a rapid protein ligation approach using a short polypeptide SpyTag and its partner, SpyCatcher derived from *Streptococcus pyogenes* fibronectin-binding protein, FbaB. To extend this technology, we have engineered and characterized a new Tag-Catcher pair from a related fibronectin-binding protein in *Streptococcus dysgalactiae*. The polypeptide Tag, named SdyTag, was constructed based on the native Cna protein B-type (CnaB) domain and was found to be highly unreactive to SpyCatcher. SpyCatcher has 320-fold specificity for its native SpyTag compared to SdyTag. Similarly, SdyTag has a 75-fold specificity for its optimized Catcher, named SdyCatcher_DANG short_, compared to SpyCatcher. These Tag-Catcher pairs were used in combination to demonstrate specific sequential assembly of tagged proteins *in vitro*. We also demonstrated that the *in vivo* generation of circularized proteins in a Tag-Catcher specific manner where specific Tags can be left unreacted for use in subsequent ligation reactions. From the success of these experiments, we foresee the application of SdyTags and SpyTags, not only, for multiplexed control of protein assembly but also for the construction of novel protein architectures.

## Introduction

Protein engineering is an important tool for many industrial, therapeutic and research applications [1]. One important aspect is fusion protein construction for applications, such as protein therapeutics (antibody fusion proteins [2, 3]), biomaterial engineering (scaffold matrices and functional groups [4]) and genetic regulation (DNA binding repressors or activators [5]). Most fusion proteins are constructed as genetically encoded proteins connected by flexible, rigid or cleavable linkers. This strategy is highly dependable on soluble expression and proper folding of the fused multi-domains constructs. To overcome solubility bottlenecks, another strategy is to assemble independently expressed and properly folded proteins *in vitro*. This strategy employs stable non-covalent interactions, such as dimerizing linkers [6], streptavidin-biotin [7], and covalent linkages such as inteins [8] and sortases [9]. While covalent *in vitro* assembly strategies bypass solubility and folding issues to form permanent linkages between proteins, some constraints of these ligation partners limit the molecular topologies that can be achieved. For example, sortases and inteins-mediated ligations can only occur between the N and C- terminus of target proteins.

Recently, a rapid covalent ligation strategy was developed by Zakeri et al [10]. This system, consisting of a peptide tag (SpyTag) and a reactive domain (SpyCatcher), was engineered by deconstructing the isopeptide forming CnaB2 domain from *S*. *pyogenes* [10]. SpyTag-SpyCatcher is an important addition to the protein engineering toolbox as it is robust, rapid and flexible in its placement within the protein. Applications for this protein ligation system have been numerous; these include controlled macromolecular assembly [11], protein cyclisation [12], cell labelling [13], antibody engineering [14] and hydrogel synthesis [15]. To realize the goal of assembling higher order macroassemblies from modular protein parts, multiple orthogonal interaction domains are needed. Using SpyTag and SpyCatcher along with other engineering strategies, this can be performed to a certain extent [11–15]. However, an orthogonal ligation partner, with SpyTag-like robustness, kinetics and flexibility, promises to significantly advance macromolecular assembly possibilities. In this study, we report the construction and optimization of a *S*. *dysgalactiae* derived Tag-Catcher pair that can be used in concert with SpyTag-SpyCatcher. The new Tag-Catcher pair will be used alongside SpyTag-SpyCatcher partners, to demonstrate kinetically controlled directed protein ligation, domain specific protein circularization and macromolecular assembly. During our preparation of this manuscript, Dr. M. Howarth at University of Oxford have also published an alternative Tag-Catcher system, SnoopTag-Catcher, that can also be utilized with SpyTag-Catcher [16].

## Materials and Methods

### Protein expression

*E*. *coli* codon-optimizated DNA sequences for Tag-Catcher constructs were synthesized using gBlocks (Integrated DNA Technologies). These were cloned into pET28 vectors using restriction enzyme digestion and ligation. The final hexahistidine-tagged constructs were transformed into T7 express *E*. *coli* (NEB) for overnight protein expression at 16°C, 100 μM isopropyl β-D-1-thiogalactopyranoside (IPTG). The cultures were centrifuged at 10000 *g* for 10 minutes at 4°C. The resulting pellets were resuspended in 25 mL of 100 mM 4-(2-hydroxyethyl)-1-piperazineethanesulfonic acid (HEPES) pH 7.4, 10 mM imidazole, 150 mM sodium chloride before sonication. The resulting lysate was then centrifuged at 19000 *g* for 1 hour at 4°C. The decanted supernatent was incubated with Ni-NTA agarose for 1 hour at 4°C. The resin was washed with 15 mL 100 mM HEPES pH 7.4, 60 mM imidazole, 150 mM sodium chloride and the bound protein was eluted with 5 mL 100 mM HEPES pH 7.4, 150 mM imidazole, 50 mM sodium chloride. The elute was diluted two fold with 100 mM HEPES pH 7.4 before loading onto a HiTrap-Q anion exchange column for FPLC (AKTA start, GE Healthcare life sciences, buffer A: 100 mM HEPES, pH 7.4, buffer B: 100 mM HEPES, pH 7.4, 1 M sodium chloride). A linear gradient elution was used. The eluted fractions were combined and buffer exchanged into 50 mM HEPES pH 7.4, 10% glycerol for storage. Catchers were expressed at ~1 mg/L yields. Tag-EGFPs were expressed at 20–30 mg/L yields. Amino acid sequences of the constructs can be found in the S1 Table.

### *In vitro* reactions

In our assay to analyze Catcher constructs, 50 μM Catcher constructs were incubated with 65 μM SdyTag-EGFP in 100 μM sodium phosphate buffer, pH 7, at 25°C. The reaction was quenched with SDS-PAGE loading buffer after 40 minutes. The samples were boiled at 95°C for 7 minutes before loading onto a 4–12% bis-tris Invitrogen precast gel. The resulting gel was stained with SimplyBlue SafeStain (Invitrogen), then imaged and analyzed using Gel Doc EZ system (Bio-Rad). Triplicates were used to measure % yield.

To measure rates, 10 μM SpyTag-EGFP was incubated with 10 μM Catcher in 100 μM sodium phosphate buffer, pH 7, at 25°C. A portion was taken out at intervals and quenched with SDS-PAGE loading buffer. The samples were boiled at 95°C for 7 minutes before loading onto a 4–12% bis-tris Invitrogen precast gel. The resulting gel was stained with SimplyBlue SafeStain, then imaged and analyzed using Gel Doc EZ. Triplicates were performed to determine rates.

For triSdyTag and bi-SdyCatcher reaction, proteins were reacted at different concentrations and different ratios for overnight in 100 μM phosphate buffer, pH 7, at 25°C. The reactions were quenched with SDS-PAGE loading buffer and boiled at 95°C for 7 minutes before loading onto a 3–8% tris-acetate Invitrogen precast gel. Further details for the specific reactions can be found in Supporting information (S1 File).

### Homology modelling

I-TASSER [17] was used to obtain a set of 5 homology models. These models were then re-assessed using the ERRAT program [18]. The model, which was chosen, had an overall quality value of 93 (percentage of the model which falls between 95% confidence level).

### Mass analysis

Mass spectrometry were performed on purified SdyCatcher_DANG short_ and purified recombinant CnaB protein (Table 1).

**Table 1:** Mass measurements.

Sample	Proteins	Expected [M+H]^+^, Da	Observed [M+H]^+^, Da	Delta mass, Da
Purified recombinant CnaB domain	Wild type self-ligated CnaB domain	16596.4	16596.8	0.4
	Gluconoylated self-ligated wild type CnaB domain	16774.4	16774.8	0.4
Purified SdyCatcher_DANG short_	SdyCatcher_DANG short_	14233.6	14233.9	0.2
	Gluconoylated SdyCatcher_DANG short_	14411.6	14412	0.3
Excess SdyTag-EGFP incubated with SdyCatcher_DANG short_	SdyTag—maturated EGFP	30633.5	30633.7	0.2
	SdyCatcher_DANG short_:N-acetylated SdyTag-EGFP (covalently ligated)	44849.1	44849.5	0.4
	Gluconoylated SdyCatcher_DANG short_: N-acetylated SdyTag-EGFP (covalently ligated)	45027.1	45026.6	-0.5

To determine if isopeptide bonds were formed, excess SdyTag-EGFP (100 μM) and SdyCatcher_DANG short_ (20 μM) was incubated overnight, in 100 mM Tris buffer, pH 6.8, at 25°C, before being subjected directly to mass spectrometry (Biopolis Shared Facilities, Singapore). Mass spectra can be found in S1 Fig. ExPASy ProtParam was accessed to calculate average mass of the proteins without N terminal methionine and using subtraction of 18 Da to account for isopeptide formation reaction. N-gluconoylation is accounted for by addition of 178 Da [19] and GFP maturation by subtraction of 20 Da [20].

### Western blot

To examine lysate fractions for TriSdyTag after slow (16°C, 100 μM IPTG, overnight) and fast expression (37°C, 100 μM IPTG, 4 hours), the cultures were centrifuged at 10000 *g* for 10 minutes at 4°C. The resulting pellets were resuspended in 25 mL of 100 mM HEPES, pH 7.4, before sonication. The lysate is then centrifuged at 19000 *g* for 1 hour at 4°C. The supernatent is then loaded onto SDS-PAGE. The proteins were separated by SDS-PAGE, were then transferred from the gel to nitrocellulose membrane and probed with Anti-6X His tag antibody (HRP, Abcam). The western blot analyses were visualized, according to manufacturer’s protocol (SuperSignal West Pico Chemilumunescent Substrate, Cat. No. 34080, Thermo Scientific).

## Results and Discussions

### Identification of a novel Tag-Catcher pair

**Fig 1:**
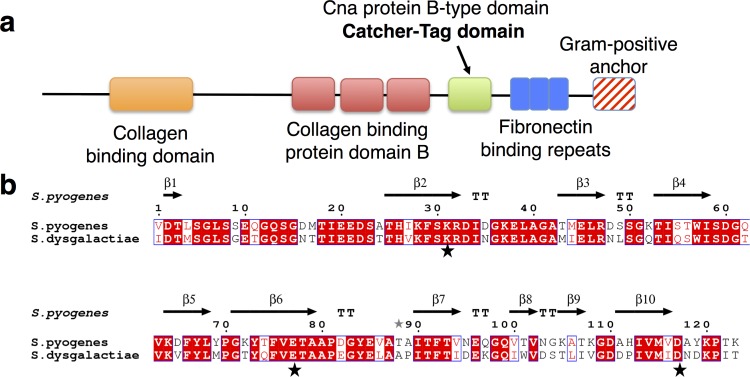
*S*. *dysgalactiae* CnaB domain analysis. (a) Graphical view of domains in *S*. *dysgalactiae* fibronectin-binding protein, accession code: CAA80122.1. This was not drawn to scale. (b) Sequence alignment of *S*. *pyogenes* and *S*. *dysgalaticae* CnaB domains using Clustal Omega [21] and ESPript 3.0 [22]. Structural beta-sheet elements (β1- β10) of the *S*. *pyogenes* protein are also indicated based on resolved *S*. *pyogenes* domain (PDB ID: 2X5P [23]). Residues involved in isopeptide formation are indicated with stars. The *S*. *dysgalactiae* CnaB domain used for alignment is located at amino acids 828–950 of the *S*. *dysgalactiae* fibronectin-binding protein (1117 amino acids long).

A BLAST search using *S*. *pyogenes* CnaB2 domain (accession code: AFD50637.1) as a query against the non-redundant protein sequences database identified a homologous CnaB protein domain from *S*. *dysgalactiae* with 63% percent sequence identity. This domain is located in a fibronectin-binding protein (accession code: CAA80122.1), consisting of an N-terminal collagen binding domain, repeat units of collagen binding protein domain B, a CnaB domain, fibronectin-binding repeats and a C-terminal cell anchoring motif (Fig 1A). Comparison between the *S*. *dysgalactiae* protein and the fibronectin-binding protein, from which SpyTag-SpyCatcher was engineered (PrtF2, accession code: AAT38844), revealed that sequence conservation is restricted to the C-terminal region containing collagen binding protein domain B, CnaB, fibronectin binding repeats and anchor domains. Sequence alignment between the *S*. *dysgalactiae* CnaB domain and its *S*. *pyogenes* counterpart showed significant conservation in the N-terminal portion (residues 1–102, Fig 1B). Sequence identity is 68% for the latter compared to 38% for the C-terminal portion (residue 103–123). Key residues involved in isopeptide formation were also conserved (Fig 1B, indicated with stars). Following the annotated boundaries of PrtF2 (accession code: CAA80122.1), a deconstructed standalone *S*. *dysgalactiae* CnaB domain was expressed in T7 Express *Escherichia coli* (New England Biolabs, NEB) with an N-terminal his-tag. Using electrospray ionization-mass spectrometry (ESI-MS) for mass determination of the purified construct, we observed masses of 16596.8 and 16774.8 Da (Table 1). These correspond, within error, to the expected average masses of a self-ligated CnaB domain with and without spontaneous *α-N*-6 gluconoylation of the N-terminal His-tag [19], 16596.4 Da and 16774.4 Da respectively. Although this demonstrates *in vivo* intra-ligation of the recombinant CnaB domain, we cannot conclude it will do the same in the native full length fibronectin binding protein. However, the intra-ligated recombinant protein indicates that a Tag-Catcher system could be engineered from the *S*. *dysgalactiae* CnaB domain.

### Catcher construction

**Fig 2:**
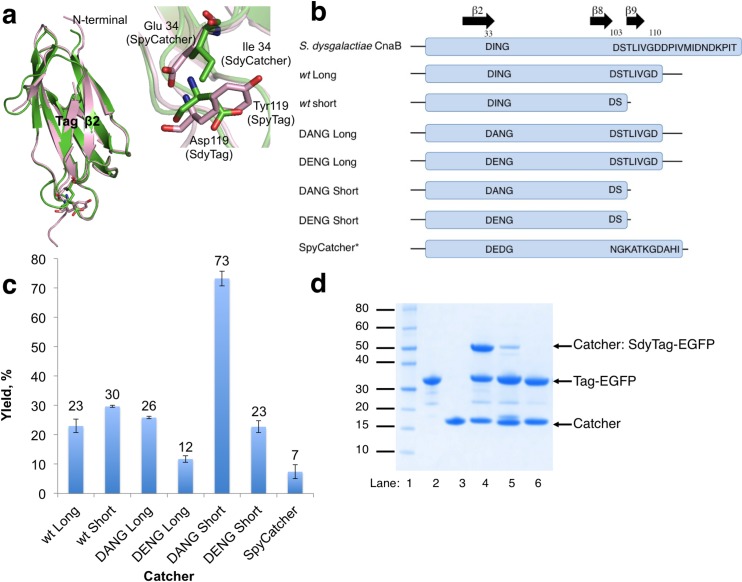
Optimization of *S*. *dysgalactiae* Catcher. (a) Alignment of I-TASSER homology model of *S*. *dysgalactiae* CnaB domain (green) and SpyTag-SpyCatcher complex (pink, 4MLI [24]). Depiction of structure was performed using PyMOL [25]. Inset depicts residues at position 119 (Tag) and 34 (Catcher) for SpyCatcher-SpyTag (pink) and SdyCatcher-SdyTag (green). (b) Schematic diagram of the Catcher variants. Amino acid sequences of the constructs can be found in S1 Table. Residue and beta sheet numbering as follows from Fig 1B. *SpyCatcher sequence as follows from Zakeri et al [10]. (c) Yield (%) of Catcher: SdyTag-EGFP product, with respect to the limiting Catcher substrate, from *in vitro* reaction of Catcher with 1.3 equivalent SdyTag-EGFP for 40 minutes at 25°C, pH 7. Averages of triplicate measurements are shown and their standard deviations are represented by error bars. Yield % are labelled. (d) Covalent ligation of SdyTag-EGFP with Catchers for 80 minutes at 25°C, pH 7. Lane 1: Novex Sharp ladder (Thermo Fisher Scientific), lane 2: SdyTag-EGFP alone, lane 3: SdyCatcher_DANG short_ alone, lane 4: SdyTag-EGFP incubated with SdyCatcher_DANG short_, lane 5: SdyTag-EGFP incubated with SpyCatcher, lane 6: SdyTag(Asp117Ala)-EGFP incubated with SdyCatcher_DANG short_.

Drawing from previous SpyCatcher structure function studies [10, 24], we dissected *S*. *dysgalactiae* CnaB domain into a Tag-Catcher pair, henceforth termed SdyTag (encoding DPIVMIDNDKPIT) and SdyCatcher respectively. To examine and optimize SdyTag-SdyCatcher interactions, we constructed and analyzed a panel of SdyCatcher variants with SdyTag. I-TASSER [17] homology modeling of the *S*. *dysgalactiae* domain yielded a model with root mean square deviation of 0.57 Å (405 atoms) to the resolved SpyTag-SpyCatcher structure (PDB: 4MLI [24]) (Fig 2A). Structural and sequence alignments of SdyCatcher with SpyCatcher were then utilized to design SdyCatcher variants (Figs 1B, 2A and 2B).

From *in silico* analyses, two main variants of SdyCatcher were designed (Fig 2B). First, we hypothesized that Ile34 (on native SdyCatcher) could interact with Glu119 (SdyTag) and thus mutations at residue position 34 (Catcher domain) could affect ligation efficiencies (Fig 2A inset). To investigate this, Catcher constructs with mutations Ile34Glu (DENG constructs) and Ile34Ala (DANG constructs) were generated, together with wild type constructs (DING constructs). DENG SdyCatcher constructs were found least efficient compared to DING and DANG constructs. DANG constructs were the most efficient (Fig 2A–2C). Yields of the short versions of DENG, DING (wild type) and DANG SdyCatcher constructs were 23, 30 and 73% respectively. These observations are likely due to the predicted electrostatic repulsion between Glu34 on DENG Catchers and Glu119 on SdyTag (Fig 2A). Isoleucine has also been suggested to inhibit Tag docking efficiencies due to its bulkier size, compared to alanine [10]. The SdyCatcher observations, where Glu substitution produced the least efficient Catcher, also differ from the previous SpyCatcher construction where an Ile34Glu mutation was shown to improve efficiencies with the Tyr119 containing SpyTag [10].

The second set of modifications consisted of removing 6 C-terminal amino acids from the Catcher construct (β9, Fig 1B). Our initial dissection point of *S*. *dysgalactiae* CnaB domain to generate the SdyTag-SdyCatcher partners, was between two aspartic acids; Asp110 (C terminal residue of SdyCatcher) and Asp111 (N terminal residue of SdyTag). To determine if this Asp (Catcher)-Asp (Tag) interaction would affect reaction rates unfavorably, C-terminal truncated constructs were made to remove the C-terminal Asp110 (long and short constructs, Fig 2B). Structural analyses of the SpyCatcher-SpyTag complex and experimental studies of C-terminus truncated SpyCatchers suggest that C-terminal portion of SpyCatcher can be removed without significant change in SpyTag ligation activity [24]. In contrast to SpyCatcher [10, 24], removal of SdyCatcher’s C-terminal portion increased ligation yields significantly (Fig 2B and 2C). Yields of DANG short and long SdyCatcher constructs were 73 and 26% respectively. Whilst the C-terminal portion of SpyCatcher was predicted to have minimal effect on Tag-Catcher ligation efficiencies [24], significant differences in yields between long and short SdyCatcher constructs indicate that the C-terminal portion of the SdyCatcher is important in Tag-Catcher ligation kinetics, possibly via inhibition of SdyTag placement onto the Catcher domain.

Overall, SdyCatcher_DANG short_ construct was considered the most efficient SdyCatcher construct (73% yield after 40 minutes reaction at 25˚C, pH 7, Fig 2C, Accession number: TBC) and was used for subsequent SdyCatcher experiments in this study. Covalent linkage of the ligated proteins was confirmed by ESI-MS of ligation reaction of SdyCatcher_DANG short_ with Sdy-EGFP (Table 1). Alanine mutation of Asp117 on the SdyTag did not yield any ligated products thus confirming the critical role of this conserved residue for isopeptide bond formation (Lane 6, Fig 2D). Reaction of SdyTag-EGFP with SpyCatcher also yielded minimal products under the same conditions (7%, Fig 2C).

### Tag-Catcher interactions

**Fig 3:**
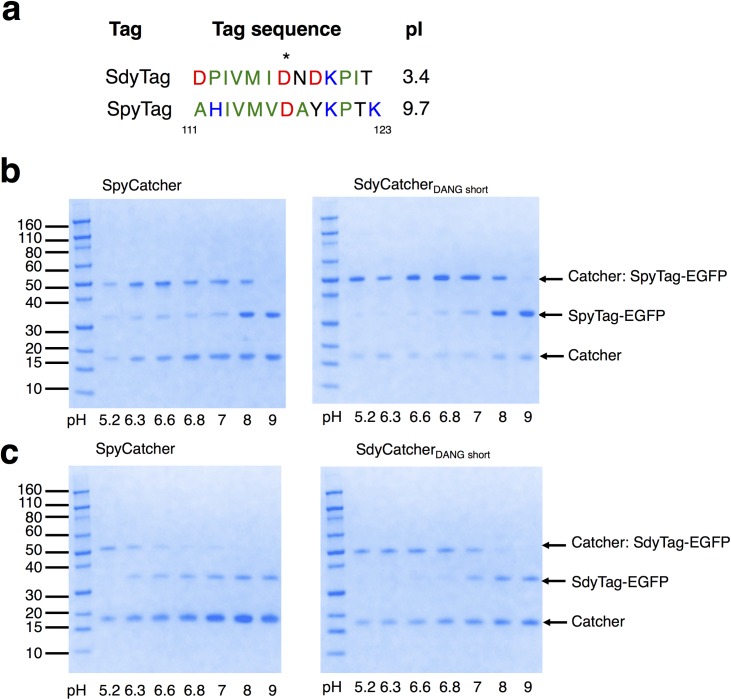
Tag variants. (a) Alignment of Tags. Hydrophobic, neutral, negatively-charged and positively-charged residues are colored as green, black, red and blue respectively. The residue numbering follows that of Fig 1B. The reactive Asp117 is indicated with a star. (b, c) pH dependence of SpyTag and SdyTag. (b) Incubation of SpyTag-EGFP with SpyCatcher (left) and SdyCatcher_DANG short_ (right) at pH 5.2, 6.3, 6.6, 6.8, 7, 8 and 9 after 10 minute at 25°C. (c) Incubation of SdyTag-EGFP with SpyCatcher (left) and SdyCatcher_DANG short_ (right) at pH 5.2, 6.3, 6.6, 6.8, 7, 8 and 9 after 30 minute at 25°C.

Based on our observations of SdyCatcher variants, we decided to investigate further Tag-Catcher interactions. Sequence alignments showed that *S*. *pyogenes* and *S*. *dysgalactiae* Catcher motifs that interact with the Tag (β2, Figs 1B and 2A) were highly similar. In contrast, despite conservation of isopeptide-forming Asp117 and hydrophobic region predicted to be necessary for Catcher interaction [24], Tags were predicted to be highly different in overall charge at pH 7 (Fig 3A). Isoelectric points (pIs) of Tags derived from *S*. *pyogenes* (SpyTag) and *S*. *dysgalactiae* (SdyTag) are predicted to be 9.7 and 3.4 respectively [26]. This difference is due to substitution of neutral residues on SpyTag with negatively charged ones on SdyTag (Ala and Tyr to Asp) and positively charged residues on SpyTag with neutral residues on SdyTag (His and Lys to Pro and Tyr respectively). To probe this further, we examined ligation of SpyTag-EGFP with SpyCatcher or with SdyCatcher_DANG short_ under various pH conditions, ranging from pH 5.2–9 (Fig 3B). Similar experiments was also performed with SdyTag-EGFP with both Catchers (Fig 3C). There are minimal changes in specificities of SpyTag for SpyCatcher versus SdyCatcher_DANG short_ with pH. SpyTag ligations were faster with lower pH (Fig 3B), similar to observations in previous SpyTag-Maltose binding protein experiments [10]. Likewise, Sdy-Tag ligation reactions were faster at lower pH. From pH 7 to 6.8, an observed doubling of yield for SdyTag-EGFP with SdyCatcher_DANG short_ was estimated based on densitometry (40% to 80% with respect to SdyTag-EGFP, Fig 3C). Catcher specificities for SdyTag-EGFP, where the SdyCatcher ligation is significantly faster compared to SpyCatcher, were also maintained up to pH 6.3. At pH 5.2, differences in yields of SdyTag-EGFP for SdyCatcher_DANG short_ and SpyCatcher were minimal (Fig 3C). This suggest that at sufficiently low pHs, interactions of SdyTag with Catchers has minimal effect on the overall ligation reaction and ligation becomes dependent on rate of isopeptide formation between the highly conserved catalytic residues in the Tag-Catcher combinations.

### Specificities of Tag-Catchers

From our observations of SdyTag with SpyCatcher (Figs 2C and 3C), we predicted that the two Tags can be utilized in combination under kinetic control. To quantify the kinetic preferences of the various Tags and Catchers, we calculated and compared second order rate constants of both SpyCatcher and SdyCatcher_DANG short_ with their non-native and native Tags fused to EGFP (Table 2). Rate constants were calculated from Catcher depletion measurements during equimolar ligation of the respective Catcher and Tag at 25°C, pH 7 (See S2–S4 Figs). The most significant observation is the 320-fold preference of SpyCatcher for its native SpyTag over SdyTag. SdyTag also has a 75-fold specificity for SdyCatcher versus SpyCatcher while SpyTag has minimal preference for SpyCatcher and SdyCatcher. Our observations of the reactivity of Catcher variants (Fig 2) in conjugation with previous studies on C-terminal truncated SpyCatcher [24], explains why SdyCatcher_DANG short_ ligate almost as efficiently with SpyTag compared to SpyCatcher. SpyTag ligation has been shown earlier to be independent of C-terminal truncations of its native Catcher [24], thus SpyTag interactions is not inhibited by both Catchers. Glu34 on the SpyCatcher would also be able to accommodate Tyr119 (SpyTag) but not Asp119 (SdyTag), while Ala34 (SdyCatcher) would be able to accomodate both Tags.

**Table 2:** Reaction rate constants between different ligation partners.

Catcher	Tag	Rate constant (M^-1^ min^-1^) 10^2^	STD (M^-1^ min^-1^) 10^2^
**Reaction with SpyCatcher**			
SpyCatcher	SpyTag	193	24
SpyCatcher	SdyTag	0.6	0.1
**Reaction with optimized SdyCatcher**			
SdyCatcher _DANG Short_	SpyTag	113	4
SdyCatcher _DANG Short_	SdyTag	45	6

Triplicate measurements were used to calculate average and standard deviations, (STD).

### Kinetic controlled Tag-Catcher ligations for directed assembly

**Fig 4:**
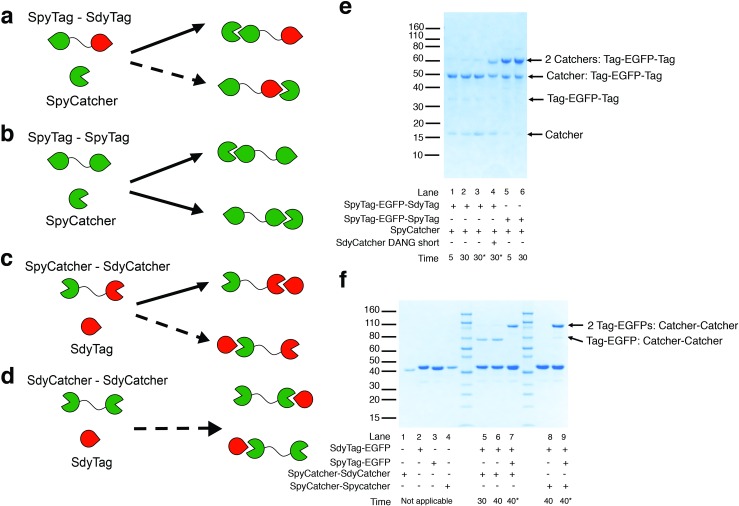
Kinetic control for directed protein assembly. Predicted preferences, based on observed reaction rates of SpyCatcher with (a) SpyTag-EGFP-SdyTag construct (preference for SpyTag versus SdyTag) compared to (b) SpyTag-EGFP-SpyTag construct (no preference for either SpyTag). Predicted preferences for SdyTag-EGFP are shown in (c) SpyCatcher-SdyCatcher_DANG short_ (preference for SdyCatcher) and (d) SpyCatcher-SpyCatcher (minimal reactivity expected). Bold arrows represent fast reaction compared to the dotted arrows which represent slow or minimal reactions. (e) Reactions of the dual-Tags constructs. Reaction of SpyTag-EGFP-SdyTag with SpyCatcher at 5 minutes (lane 1) and 30 minutes (lane 2). At 5 minutes, aliquots were removed and incubated with additional SpyCatcher (lane 3) or additional SdyCatcher_DANG short_ (lane 4) for a further 25 minutes. Reaction of SpyTag-EGFP-SpyTag with SpyCatcher at 5 minutes (lane 5) and 30 minutes (lane 6). (f) Reactions of the dual-Catcher constructs. Lane 1–4 are standalone purified SpyCatcher- SdyCatcher_DANG short_, SdyTag-EGFP, SpyTag-EGFP, SpyCatcher-SpyCatcher respectively. Incubation of SpyCatcher- SdyCatcher_DANG short_ with excess SdyTag-EGFP for 30 and 40 minutes (lanes 5 and 6). An aliquot of the reaction after 30 minutes was removed and incubated with excess SpyTag-EGFP for further 10 minutes (lane 7). Reaction of SpyCatcher-SpyCatcher with excess SdyTag-EGFP for 40 minutes (lane 8). Excess SpyTag-EGFP was incubated with an aliquot of the latter reaction, after 30 minutes, for a further 10 minutes (lane 9). Astericks indicate the reactions which additional substrates were incubated in the reactions. Novex Sharp pre-stained ladder was used in both gels.

Comparison of rate constants between Catcher-Tag partners (Table 2) suggests that directed ligation schemes can be generated under two conditions. First, the 320-fold preference of SpyCatcher for its native SpyTag over SdyTag can be used to select for SpyTag in a mixture of Tags (Fig 4A and 4B). Second, 75-fold preference of SdyTag with SdyCatcher_DANG short_ over SpyCatcher can be utilized to select for SdyCatcher_DANG short_ in a mixture of Catchers (Fig 4C and 4D). Directed protein assembly is also performed with the considerations that SdyCatcher does not demonstrate preference for SdyTag over SpyTag and that SpyTag can also react almost as efficiently with both Catchers.

To apply the Tags for directed protein ligation, dual Tagged or dual Catcher proteins were constructed. A dual SpyTag-EGFP-SdyTag (Fig 4A), along with its SpyTag only equivalent (SpyTag-EGFP-SpyTag, Fig 4B) were designed and expressed. Similarly, a dual Catcher, consisting of SpyCatcher-SdyCatcher_DANG short_ (Fig 4C), and its SpyCatcher only equivalent (SpyCatcher-SpyCatcher, Fig 4D) were constructed and expressed.

To evaluate selectivity of each Catcher, the dual-Tag construct was used to ligate specific Catcher constructs in a step-wise *in vitro* reaction (Fig 4E). We first incubate the dual-Tag with SpyCatcher to obtain the specifically ligated SpyCatcher:SpyTag-EGFP-SdyTag. As predicted, with the high preference of SpyCatcher for SpyTag over SdyTag, we observed only one major product with the incubation of SpyCatcher with dual-Tag (lanes 1–3, Fig 4E). This product is predicted to be SpyCatcher:SpyTag-EGFP-SdyTag, where SdyTag is unreacted. Under these conditions, there is also little cross-reactivity between Tag-Catcher partners as we observe minimal double SpyCatcher ligated product (SpyCatcher:SpyTag-EGFP-SdyTag:SpyCatcher). Based on densitometry, less than 10% of the products were ligated non-specifically twice (with a protein product of 60 kDa, lanes 1–2, Fig 4E). With the further addition of SdyCatcher_DANG short_, the double-ligated product (SpyCatcher:SpyTag-EGFP-SdyTag:SdyCatcher) is observed (lane 4, Fig 4E). In contrast, addition of SpyCatcher did not result in any change in protein profile (lane 3, Fig 4E). In the SpyTag-only equivalent, the sequential ligation reaction was not possible, where both singly and doubly ligated SpyTag-EGFP-SpyTag products were observed under the same conditions (lanes 5 and 6, Fig 4E).

Next, we demonstrate directed ligation using SpyCatcher-SdyCatcher_DANG short_ construct (Fig 4F). Upon incubation of SpyCatcher-SdyCatcher_DANG short_ with SdyTag-EGFP, we observed the appearance of a major protein band corresponding to a singly ligated product (60 kDa, lanes 5 and 6, Fig 4F). From the rate constants, this is predicted to be SpyCatcher-SdyCatcher_DANGshort_:SdyTag-EGFP. Based on densitometry, <10% of the products were ligated non-specifically twice (90 kDa, lanes 5 and 6, Fig 4F). Only the further addition of SpyTag-EGFP, produced the predicted SpyTag-EGFP:SpyCatcher-SdyCatcher_DANG short_:SdyTag-EGFP (90 kDa, lane 7, Fig 4F) after 10 minutes. In comparison, minimal product was observed when SpyCatcher-SpyCatcher was reacted with SdyTag-EGFP after 40 minutes under the same conditions (Lane 8, Fig 4F). Together, these results demonstrate that SdyTag-EGFP can be directed onto SpyCatcher- SdyCatcher_DANG short_ to produce SpyCatcher- SdyCatcher_DANG short_:SdyTag-EGFP, which can be, in turn, ligated further with SpyTag-EGFP.

Utilizing the selectivities of SpyCatcher and SdyTag, we have demonstrated that under kinetic control, one-pot directed protein ligation is possible. Although, our current assembly was accomplished with EGFP fusions, we do not foresee any difficulty fusing other proteins onto the Tag-Catchers as shown from previous studies [11, 12, 15]. To further enhance yields, solid phase addition, as demonstrated by SnoopTag [16], or step-wise purifications could also be utilized.

### Construction and polymerization of circular proteins

Next, we also demonstrated utility of the Tag-Catcher pairs for macromolecular control. Although, circular proteins have been constructed previously using SpyTag-SpyCatcher [11, 12], development of the SdyTag presents us with an opportunity to generate specific internally-circularized proteins with specific free Tags to be used for further protein assembly possibilities.

**Fig 5:**
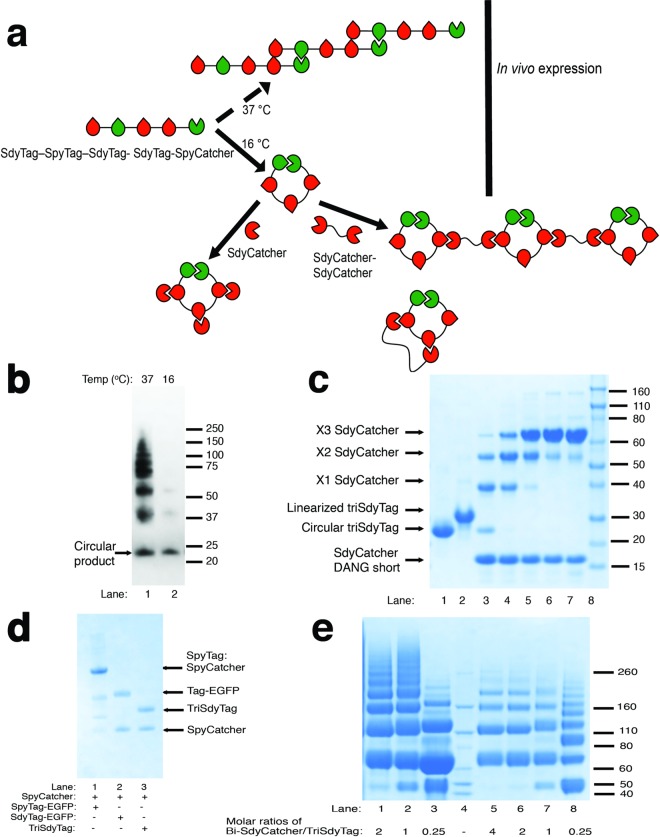
Construction and polymerization of circular proteins. (a) Schematics for *in vivo* and *in vitro* ligations of triSdyTag constructs. (b) Western blot showing *E*.*coli* lysates for the production of triSdyTag with 100 μM IPTG, 16°C (lane 2) and 37°C (lane 1). (c) Characterization of triSdyTag. Lane 1: Purified triSdyTag, lane 2: purified triSdyTag after TEV digestion overnight, lanes 3–7: reaction of SdyCatcher_DANG short_ and triSdyTag at 1, 5, 20, 90 minutes and overnight at 25°C, pH 7 and lane 8: Novex Sharp ladder. (d) Presence of free SpyTag was examined by reaction of SpyCatcher with either SpyTag-EGFP (lane 1), SdyTag-EGFP (lane 2) or circularized triSdyTag (lane 3) at 5 minutes, pH 7, 25°C. Only SpyTag-EGFP (lane 1) has a product (SpyTag-EGFP:SpyCatcher) with SpyCatcher under these conditions. (e) Polymerization of tri-SdyTag. Lanes 1–3: Reaction of bi-SdyCatcher with triSdyTag at 30 μM in 2:1, 1:1, 1:4 Catcher: Tag molar ratios, lane 4: Novex Sharp ladder and lanes 5–8: reaction of bi-SdyCatcher with triSdyTag at 10 μM in 4:1, 2:1, 1:1, 1:4 Catcher: Tag molar ratios. The reactions were left overnight at 25°C, pH 7.

From the observed rate constants (Table 2) and kinetic controlled Tag-Catcher assembly results (Fig 4), SpyCatcher has demonstrated preference for its native SpyTag in the presence of both SpyTag and SdyTag *in vitro*. We hypothesize that the same selection should occur *in vivo*. To demonstrate this, we designed a multi-Tag construct. This multi-Tag construct consist of 3 SdyTags, 1 SpyTag and 1 SpyCatcher, arranged as SdyTag-SpyTag-SdyTag-SdyTag-tobacco etch virus (TEV) protease site-SpyCatcher (triSdyTag, Fig 5A). We predicted that SpyTag-SpyCatcher ligation would proceed rapidly compared to SdyTag-SpyCatcher ligation, subsequently, *in situ* intra-polypeptide ligation, under slow expression conditions, is expected to mainly produce the SpyTag-SpyCatcher ligated circularized protein (Fig 5A).

The triSdyTag construct were expressed in T7 Express *E*. *coli* at 100 μM IPTG under 16°C overnight (slow expression) or 37°C for 4 hours (fast expression). The cell pellets were lysed and their protein profiles were examined by Western Blot analysis with an anti- 6xHistag antibody (HRP, Abcam) (Fig 5B). Not surprisingly, multi-mer proteins were observed at 37°C whilst the 16°C expression yielded a primary protein band at ~20 kDa (77% of the lysate based on densitometry). TriSdyTag is predicted to have a mass of 22 kDa.

Using Ni-NTA purification and followed by fast protein liquid chromotagraphy (FPLC), ~20 kDa sized proteins were purified from 16°C expression of triSdyTag (lane 1, Fig 5C). Circularization of the triSdyTag product was confirmed with TEV protease digestion, where a single band of lower mobility was observed after overnight digestion (lanes 1 and 2, Fig 5C). We further verified presence of three accessible Tags on the triSdyTag with a maximum addition of three SdyCatchers onto tri-SdyTag (lanes 3–8, Fig 5C). To determine if the circularization was specific (SpyTag to SpyCatcher), we used SpyCatcher ligation as a means to determine the percentage of free SpyTag present in the triSdyTag. TriSdyTag had no observable product with SpyCatcher (lane 3, Fig 5D). This was similar to the reaction of SdyTag-EGFP with SpyCatcher, which had no product under the same conditions (lane 2, Fig 5D). Thus, this suggests that there are minimal free SpyTag in the circular protein and subsequently, also verifies that the *in situ* protein circularization took place specifically via SpyTag and SpyCatcher. In the presence of a 3:1 SdyTag: SpyTag population and under slow expression conditions, we have demonstrated *in vivo* selectivity of the SpyCatcher for its native Tag and the construction of a circularized protein with accessible internal and N-terminal SdyTags.

Besides ligating the circular triSdyTag with SdyCatcher fusions, a triSdyTag could be utilized to form a protein network, similar to the Spy hydrogel network created using tetraSpyTag with double SpyCatcher containing constructs [15]. In this assembly, the network would be formed between circular proteins instead of linear Tag-containing proteins. A bi-SdyCatcher “connector” was constructed (SdyCatcher_DANG short_—SdyCatcher_DANG short_, Fig 5A) and added to the triSdyTag *in vitro* (Fig 5E). With excess TriSdyTag, an additional set of N-mers were observed compared to excess or equimolar of bi-SdyCatchers (Fig 5E). To determine the maximum size of the protein assembly, we also ran the 2:1 Catcher:Tag reaction (lane 1, Fig 5E) on a size exclusion chromatography column, Tosoh TSKgel G3000SWxl, which has a calibrated range of 10–500 kDa for globular proteins (S5 Fig). The maximum size observed was > 500 kDa.

## Conclusions

In this study, we engineered and characterized a new protein ligation Tag-Catcher pair, SdyTag-SdyCatcher. Like SpyTag-Catcher, protein ligation occurs via the formation of an isopeptide bond. We showed that SdyTag-SdyCatcher ligations can also occur between N, C-terminus and internal sites under various pH conditions. We took advantage of the unreactive nature of SdyTag for SpyCatcher to perform kinetic controlled directed protein assembly *in vivo* and *in vitro*. Based on analyses of EGFP-fused Tags, SpyCatcher has a 320-fold preference for its native SpyTag compared to SdyTag. SdyTag also has a 75-fold preference for SdyCatcher_DANG short_ compared to SpyCatcher. Although our kinetics were performed using EGFP-fused Tags and might not be applicable for all fusion proteins, selectivity between Tags have also been demonstrated using a standalone multiple Tag-SpyCatcher system (Fig 5).

Using dual-Tag (SpyTag-EGFP-SdyTag) and dual-Catcher (SpyCatcher-SdyCatcher) constructs, we have demonstrated methods for directed protein ligation *in vitro*. Although our assembly was accomplished “linearly”, we expect that SdyTag can also be used for macromolecular assembly of proteins in the many other unique architectures demonstrated previously with the SpyTag-SpyCatcher ligation strategy (e.g. star-shaped, H-arms [11]). The ability to control the order of proteins assembled would also be particularly advantageous towards assembling a specific sequence of protein domains, in particular for biosynthetic pathways [27].

Addition of the SdyTag-SdyCatcher to the protein ligation toolbox also presented an opportunity to re-examine protein circularization. Circularized proteins are advantageous for their thermal stability and resistance to degradation [28]. Previous strategies for end-to-end circularization include chemical ligation and sortases [28]. With the SpyTag-SpyCatcher alone, domain specific circularization has been demonstrated by Arnold et al [11]. Using both SdyTag and SpyTag in this study, specifically circularized proteins with free SdyTags, which can be further used for *in vitro* macromolecular assembly, can be expressed *in vivo*. In addition to the circular construct in this study, we anticipate the *in vivo* specificity of the Tag-Catchers would be an attractive ability in generating another dimension to un-natural protein scaffolds.

In our characterization and engineering of the SdyTag-SdyCatcher, we managed to demonstrate directed protein assembly using SdyTag and SpyTag, however, we recognize that the current capabilities of SdyTag-SdyCatcher is limited due to SdyTag’s eventual cross-reactivity to SpyCatcher. Despite this, our optimisation experiments have highlighted importance of different Tag-Catcher interactions that could be potentially used in multiplexing protein ligations.

## Supporting Information

S1 Fig Deconvoluted electrospray ionization- mass spectrometry (ESI-MS) spectra. Spectra of (a) the standalone *S*. *dysgalactiae* CnaB domain, (b) SdyCatcher DANG short alone and of (c, d) incubation of excess SdyTag-EGFP with SdyCatcher DANG short.
(TIFF)

S2 Fig Yield (%) of ligated SpyTag product with time (minutes). SpyTag-EGFP, 10 μM, is incubated with with SdyCatcher (10 μM, diamond) and SpyCatcher (10 μM, square).
(TIFF)

S3 Fig Yield (%) of ligated SdyTag product with time (minutes). SdyTag-EGFP, 50 μM, is incubated with (a) SdyCatcher (50 μM) and (b) SpyCatcher (50 μM).
(TIFF)

S4 Fig Rate constant for reactions. SpyTag-EGFP with (a) SpyCatcher and (b) SdyCatcher at 10 μM concentrations. SdyTag-EGFP with (c) SpyCatcher and (d) SdyCatcher at 50 μM concentrations. Trend line equations and coefficients are shown.
(TIFF)

S5 Fig Size exclusion column chromotagraphy of 2:1 bi-SdyCatcher to triSdyTag ligation reaction. The elution time for the different mass standards are also annotated. Indicated peaks have the masses: (1) >669 kDa, (2) ~300 kDa, (3) ~210 kDa, (4) ~150 kDa, (5) ~100 kDa and (6) <30 kDa.
(TIFF)

S1 File Methods for *in vitro* reactions and size exclusion chromatography. (DOCX)

S1 Table Protein sequences of constructs used in the study. (XLSX)
