# Supplementary figures and images for: Kinetic Controlled Tag-Catcher Interactions for Directed Covalent Protein Assembly
Source: PLoS One. 2016 Oct 26;11(10):e0165074. doi: 10.1371/journal.pone.0165074 (PMC5082641; doi:10.1371/journal.pone.0165074)

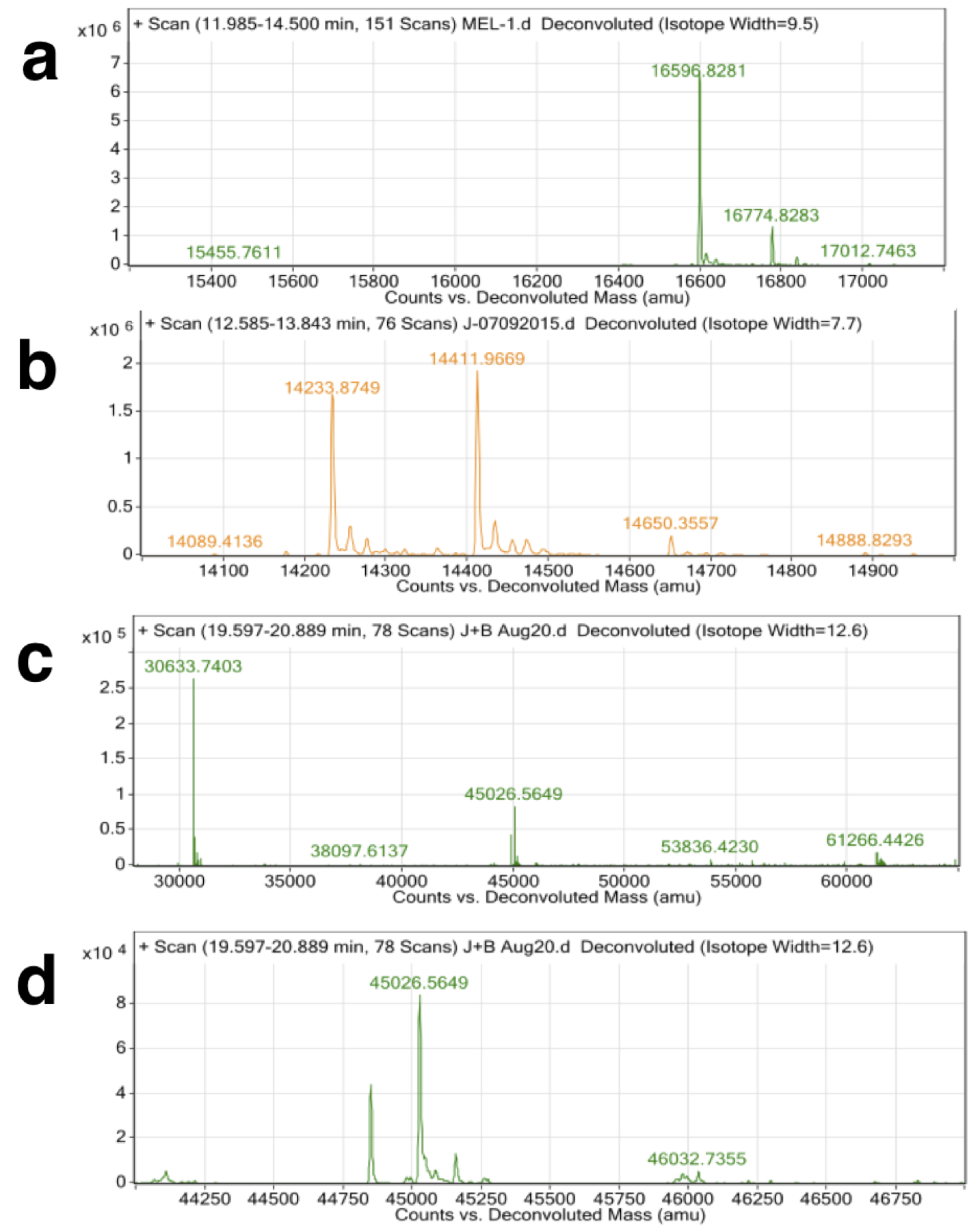

Supplement: S1 Fig — Spectra of (a) the standalone S. dysgalactiae CnaB domain, (b) SdyCatcher DANG short alone and of (c, d) incubation of excess SdyTag-EGFP with SdyCatcher DANG short. (TIFF) [file pone.0165074.s001.tiff]

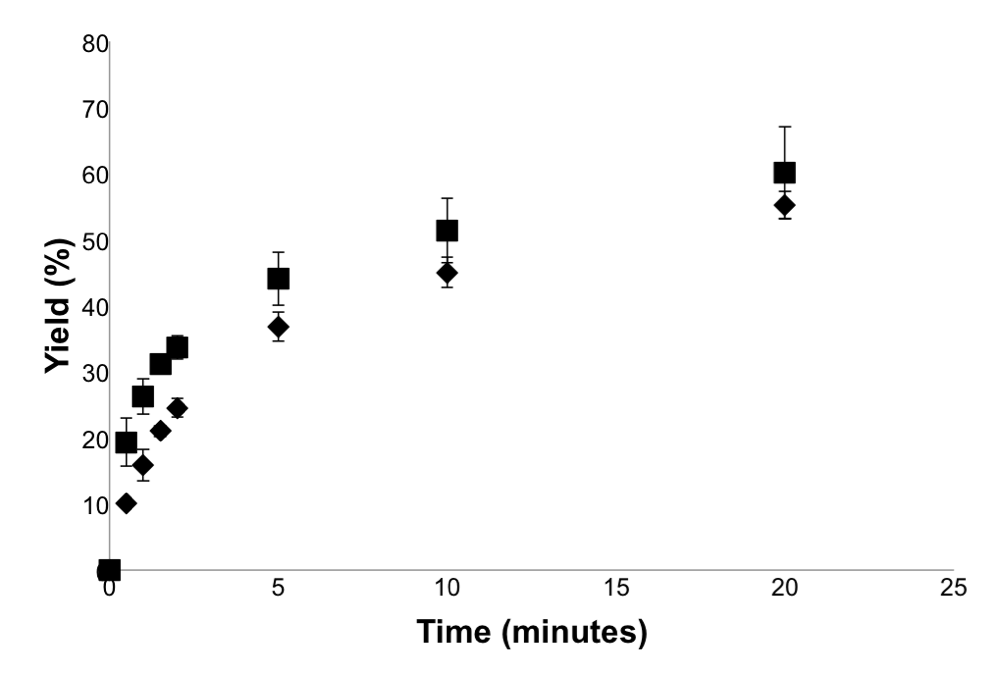

Supplement: S2 Fig — SpyTag-EGFP, 10 μM, is incubated with with SdyCatcher (10 μM, diamond) and SpyCatcher (10 μM, square). (TIFF) [file pone.0165074.s002.tiff]

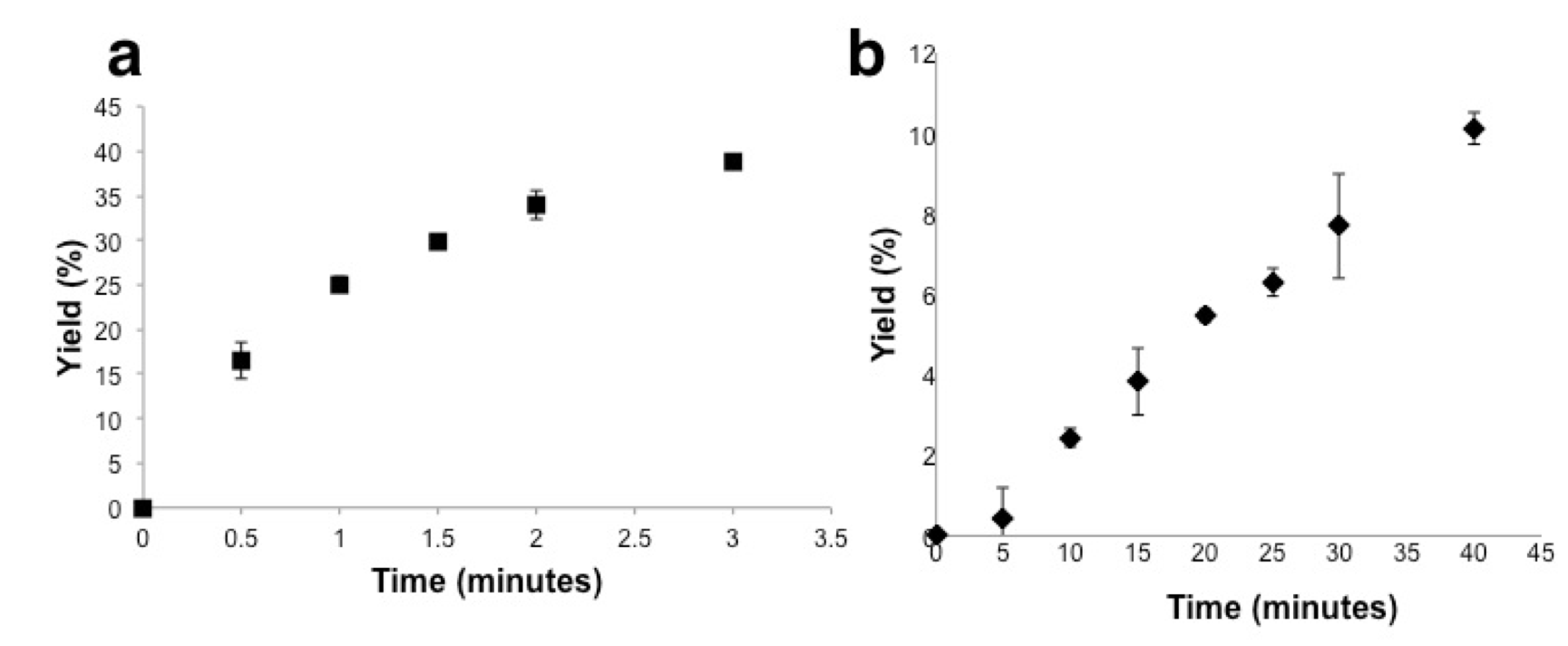

Supplement: S3 Fig — SdyTag-EGFP, 50 μM, is incubated with (a) SdyCatcher (50 μM) and (b) SpyCatcher (50 μM). (TIFF) [file pone.0165074.s003.tiff]

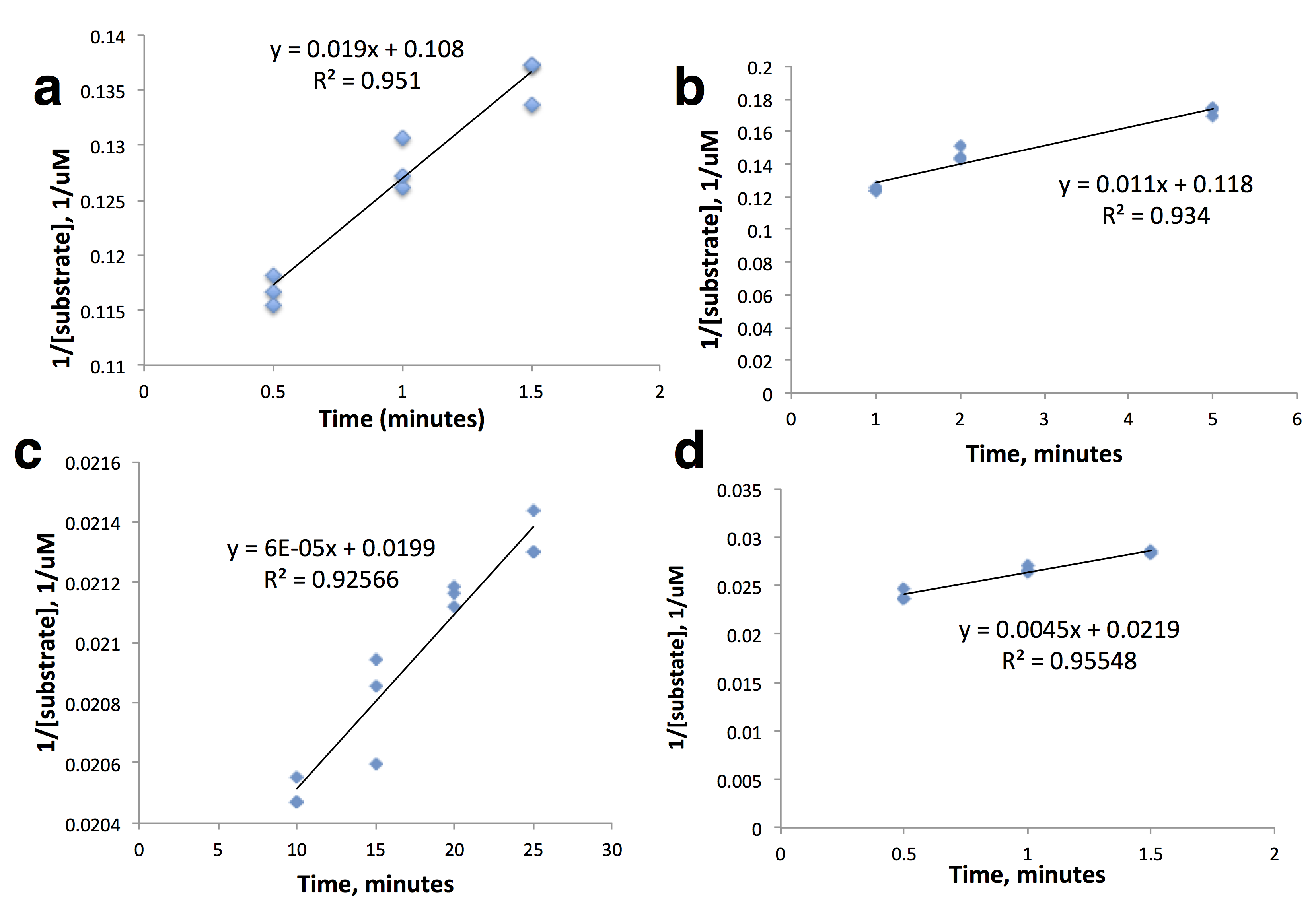

Supplement: S4 Fig — SpyTag-EGFP with (a) SpyCatcher and (b) SdyCatcher at 10 μM concentrations. SdyTag-EGFP with (c) SpyCatcher and (d) SdyCatcher at 50 μM concentrations. Trend line equations and coefficients are shown. (TIFF) [file pone.0165074.s004.tiff]

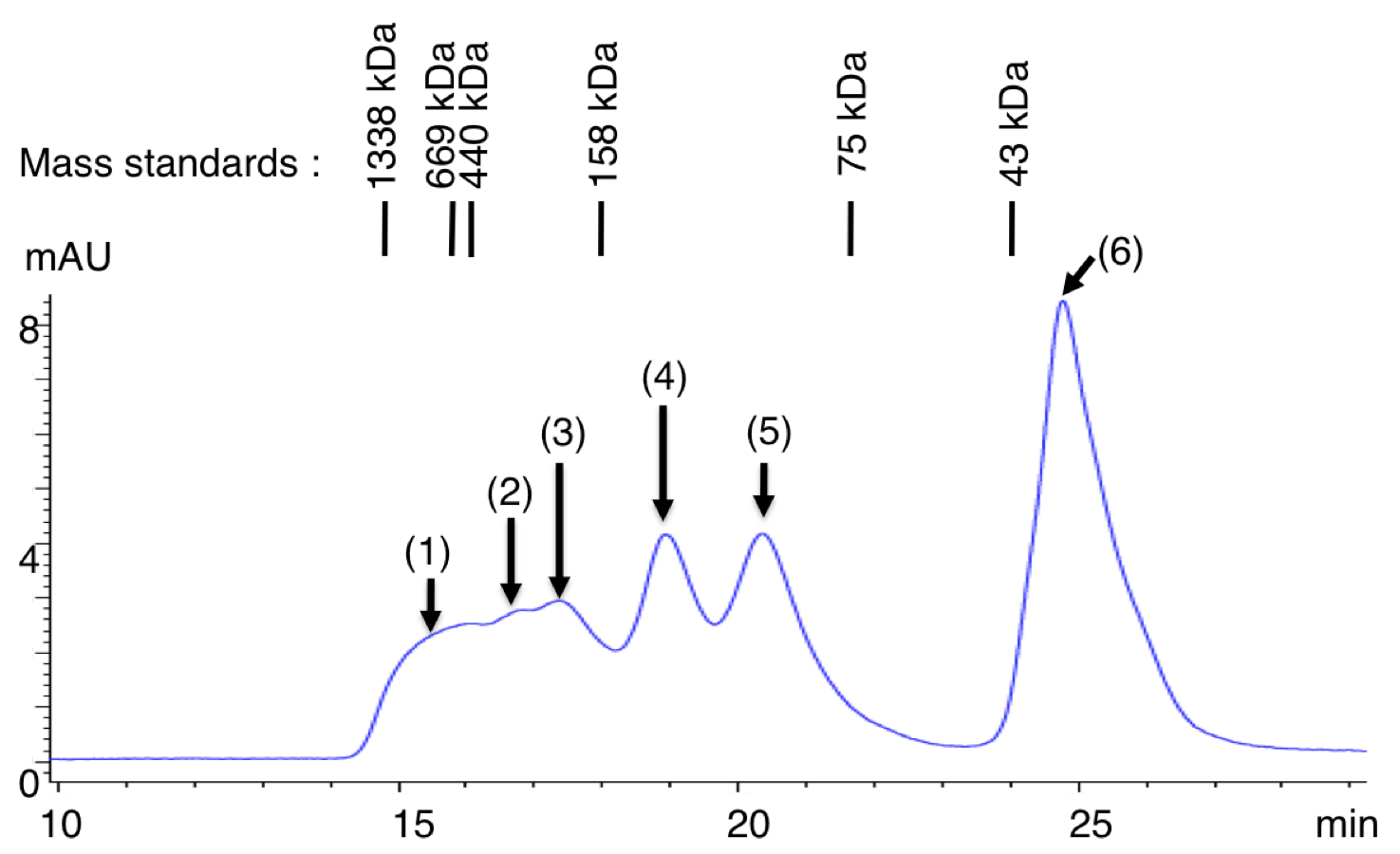

Supplement: S5 Fig — The elution time for the different mass standards are also annotated. Indicated peaks have the masses: (1) >669 kDa, (2) ~300 kDa, (3) ~210 kDa, (4) ~150 kDa, (5) ~100 kDa and (6) <30 kDa. (TIFF) [file pone.0165074.s005.tiff]
