# Supplementary material for: Kinetic Controlled Tag-Catcher Interactions for Directed Covalent Protein Assembly
Source: PLoS One. 2016 Oct 26;11(10):e0165074. doi: 10.1371/journal.pone.0165074 (PMC5082641; doi:10.1371/journal.pone.0165074)
Supplement: S1 File — (DOCX) [file pone.0165074.s006.docx]

**Supporting information**

**Kinetic controlled Tag-Catcher interactions for directed covalent protein assembly**

Lee Ling, Tan^1^, Shawn S., Hoon^1^, Fong T., Wong^1*^

^1^Molecular Engineering Lab, Biomedical Sciences Institutes, 61 Biopolis Drive, Singapore 138673, Singapore.

**Methods**

***In vitro* reactions**

**Figure 2c:** In our initial assay to analyze Catcher constructs, 50 μM Catcher constructs were incubated with 65 μM SdyTag-EGFP, 100 mM sodium phosphate buffer, pH 7. The reactions were quenched with SDS-PAGE loading buffer after 40 minutes. % yield were measured in triplicates.

**Figure 2d:** To demonstrate specificity of SdyTag for SdyCatcher_DANG short_ over SpyCatcher, 50 μM SdyTag-EGFP or 50 μM SdyTag (Asp117Ala)-EGFP was incubated with 50 μM Catcher in 100 mM sodium phosphate buffer, pH 7. The reactions were quenched with SDS-PAGE loading buffer after 80 minutes.

**Figure 3b, c:** To determine pH dependence, 10 μM SpyTag-EGFP or 50 μM SdyTag-EGFP was incubated with 2 equivalent Catcher for 1 min for SpyTag-EGFP or 30 mins for SdyTag-EGFP in 100 mM buffers of various pH (sodium acetate pH 5.2, Tris pH 6.3, Tris pH 6.6, Tris pH 6.8, Tris pH 7.0, Tris pH 8.0, Tris pH 9.0). The reactions were quenched with SDS-PAGE loading buffer.

**Figure 4e:** In SpyTag-EGFP-SdyTag (20 μM) with SpyCatcher (40 μM) reaction, proteins were reacted for between 5 - 30 minutes, in 100 mM Tris buffer, pH 6.8. An aliquot of the reaction was removed at 5 minutes and either SpyCatcher or SdyCatcher_DANG short_ was added and quenched after a further 25 minutes. The reactions were quenched with SDS-PAGE loading buffer.

In SpyTag-EGFP-SpyTag (20 μM) with SpyCatcher (40 μM) reaction, proteins were incubated for 5 and 30 minutes, in 100 mM Tris buffer, pH 6.8. The aliquots were quenched with SDS-PAGE loading buffer. The samples were loaded onto a 4-15% Mini-PROTEAN TGX gel (Bio-Rad).

**Figure 4f:** Dual-Catcher (SpyCatcher- SdyCatcher_DANG short_: 50 μM, SpyCatcher-SpyCatcher: 50 μM) was incubated with excess SdyTag-EGFP (100 μM) reaction for 30-40 mins, in 100 mM Tris buffer, pH 6.8. SpyTag-EGFP (100 μM) was added to aliquots of both reactions at 30 minutes and these were quenched after a further 10 minutes. The reactions were quenched with SDS-PAGE loading buffer.

**Figure 5c:** For the ligation of triSdyTag and SdyCatcher_DANG short_, 50 μM of triSdyTag was incubated with 150 μM of SdyCatcher_DANG short_ in 100 mM sodium phosphate buffer, pH 7. A portion was taken out at intervals (1, 5, 20, 90 minutes and after overnight incubation) and quenched with SDS-PAGE loading buffer.

**Figure 5d:** To measure the presence of free SpyTag, 10 μM of either SpyTag-EGFP, SdyTag-EGFP, or circularized triSdyTag was incubated with 10 μM SpyCatcher at 5 mins, 100 mM sodium phosphate buffer, pH 7. The reactions were quenched with SDS-PAGE loading buffer.

**Figure 5e:** For triSdyTag and bi-SdyCatcher reaction, proteins were reacted at different concentrations and different ratios for overnight, 100 mM phosphate buffer, pH 7. The reactions were quenched with SDS-PAGE loading buffer. The samples were loaded onto a NuPAGE Novex 3-8 % Tris-Acetate gel (Thermo Fisher Scientific).

**Table 2:** To measure rates, 10 μM SpyTag-EGFP was incubated with 10 μM Catcher in 100 mM sodium phosphate buffer, pH 7. Aliquots were removed at intervals and quenched with SDS-PAGE loading buffer. 50 μM SdyTag-EGFP was incubated with 50 μM Catcher in 100 mM sodium phosphate buffer, pH 7. Aliquots were removed at intervals and quenched with SDS-PAGE loading buffer. % yield and substrate concentration were measured in triplicates. (see S2 - S4 Figs)

Unless mentioned otherwise, reactions were performed at 25 °C and after quenching with SDS-PAGE loading buffer, samples were boiled at 95 °C for 10 minutes before loading onto a NuPAGE Novex 4-12% Bis-Tris gel (Thermo Fisher Scientific) with 2-(N-morpholino)ethanesulfonic acid (MES) SDS running buffer. The resulting gel was stained with SimplyBlue SafeStain (Thermo Fisher Scientific), then imaged and analyzed using Gel Doc EZ system (Bio-Rad).

**Size exclusion chromatography**

For size exclusion chromatography, protein samples were ran on Tosoh TSKgel G3000SWxl in phosphate-buffered saline (PBS) at 0.4 mL/min at room temperature. UV absorption of 280 nm was used for detection.
